# Long-Term Outcomes of Balloon Dilation for Acquired Subglottic Stenosis in Children

**DOI:** 10.1155/2014/304593

**Published:** 2014-02-19

**Authors:** Aliye Filiz, Seckin O. Ulualp

**Affiliations:** Department of Otolaryngology-Head and Neck Surgery, Division of Pediatric Otolaryngology, University of Texas Southwestern Medical Center and Children's Medical Center, 5323 Harry Hines, Dallas, TX 75390-9035, USA

## Abstract

*Objectives*. Balloon dilation laryngoplasty has been suggested as an alternative treatment to open surgical treatment of acquired subglottic stenosis in children. We describe long-term outcomes of balloon dilation for acquired subglottic stenosis in children. *Methods*. The medical charts of children who had balloon dilation for subglottic stenosis secondary to intubation were reviewed. Data included demographics, relevant history and physical examination, diagnostic workup, and management. Outcomes of balloon dilation were assessed based on improvement in preoperative symptoms, grading of stenosis, complications, and need for additional procedures. *Results*. Three children (2 male, 1 female, age range: 14 weeks–1 year) underwent balloon dilation for acquired subglottic stenosis. Patients presented with stridor and increased work of breathing. Duration of intubation ranged from 2 days to 3 weeks. Patients became symptomatic 5 days to 6 weeks after extubation. Grade of subglottic stenosis was II in 2 patients and III in one. Subglottic stenosis patients had 2-3 dilations within 2–10 weeks. All patients were asymptomatic during 14–21-month follow-up. *Conclusions*. Serial balloon dilation was safe and successful method to manage acquired subglottic stenosis in this group of children. No recurrence was noted in a follow-up more than a year after resolution of symptoms.

## 1. Introduction

Acquired subglottic stenosis in children has been treated using a wide variety of approaches such as observation, dilation, laser, single or multistage open surgical reconstructions, and tracheostomy. With the advent of high-pressure, noncompliant airway balloons, balloon laryngoplasty has been increasingly used for treatment of acquired subglottic stenosis in children. Balloon dilation of the airway provides an opportunity to apply radial force to subglottic narrowing and prevents shearing forces created by rigid dilators [1]. Serial balloon dilations may be needed and open procedures may be avoided in some patients. Outcomes of balloon dilation in children have been increasingly investigated. Nevertheless, long-term outcomes of balloon dilation for acquired subglottic stenosis have not been established in children. The aim of the present study is to describe long-term outcomes of balloon dilation for acquired subglottic stenosis in children.

## 2. Case Reports

The medical charts of children who had balloon dilation for subglottic stenosis secondary to intubation were reviewed. Data included demographics, relevant history and physical examination, diagnostic workup, and management. Outcomes of balloon dilation were assessed based on improvement in preoperative symptoms, grading of stenosis, complications, and need for additional procedures.

Three children (2 males, 1 female, age range: 14 weeks–1 year) underwent balloon dilation for management of subglottic stenosis. The degree of subglottic stenosis was endoscopically graded before and after balloon dilations using the Myer-Cotton classification [2]. Balloon dilation technique included insertion of balloon catheter to the stenotic region under direct telescopic visualization. Then the balloon was inflated to a pressure of 2 atm. Balloon pressure was maintained for 30 seconds or until the patient's oxygen saturation dropped to 92%. The airway was reassessed using the telescope and an endotracheal tube was inserted into the dilated airway for oxygenation and sizing the airway. Balloon dilation was performed 3 times during each session. Patients did not need endotracheal intubation after balloon dilation. All patients received 5 days of systemic corticosteroid (prednisolone, 1-2 mg/kg/d) and acid suppressive treatment for 4 weeks. No complications occurred.

**Figure 1:**
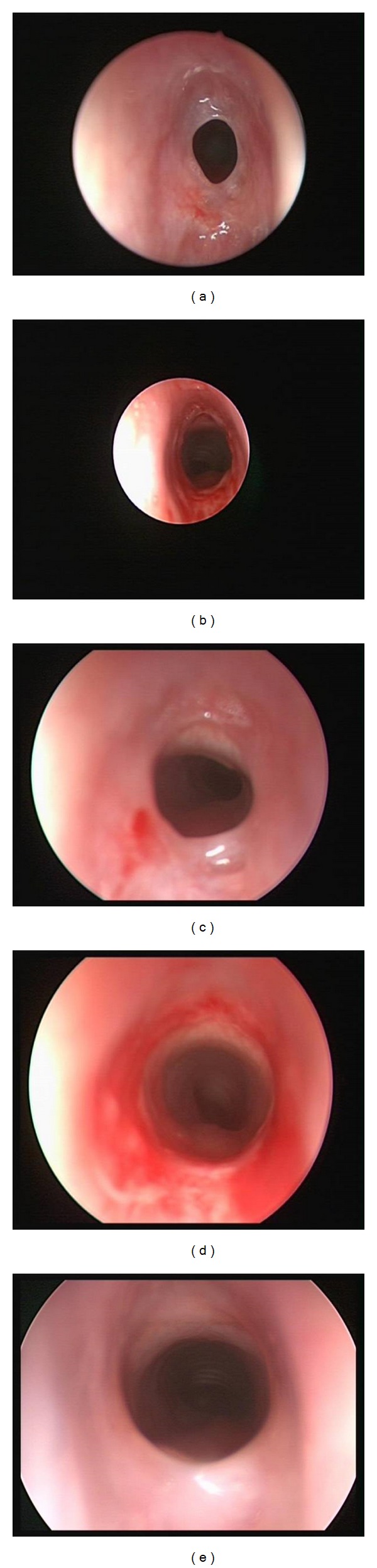
Pre- and postdilation images of subglottic stenosis at the initial dilation ((a) and (b)), second dilation ((c) and (d)), and 14-month follow-up (e).

Patient 1 was 12-month-old female who required intubation for 2 days after she presented with acute liver failure. Upon extubation, she developed stridor and increased work of breathing. Airway examination documented Grade III, soft subglottic stenosis and balloon dilation was performed. Repeat balloon dilations were performed at 2 weeks and 4 weeks after the initial balloon dilation (Figure 1). The airway was stable 3 months after the first balloon dilation. The child has been followed for 14 months with no stridor or increased work of breathing.

**Figure 2:**
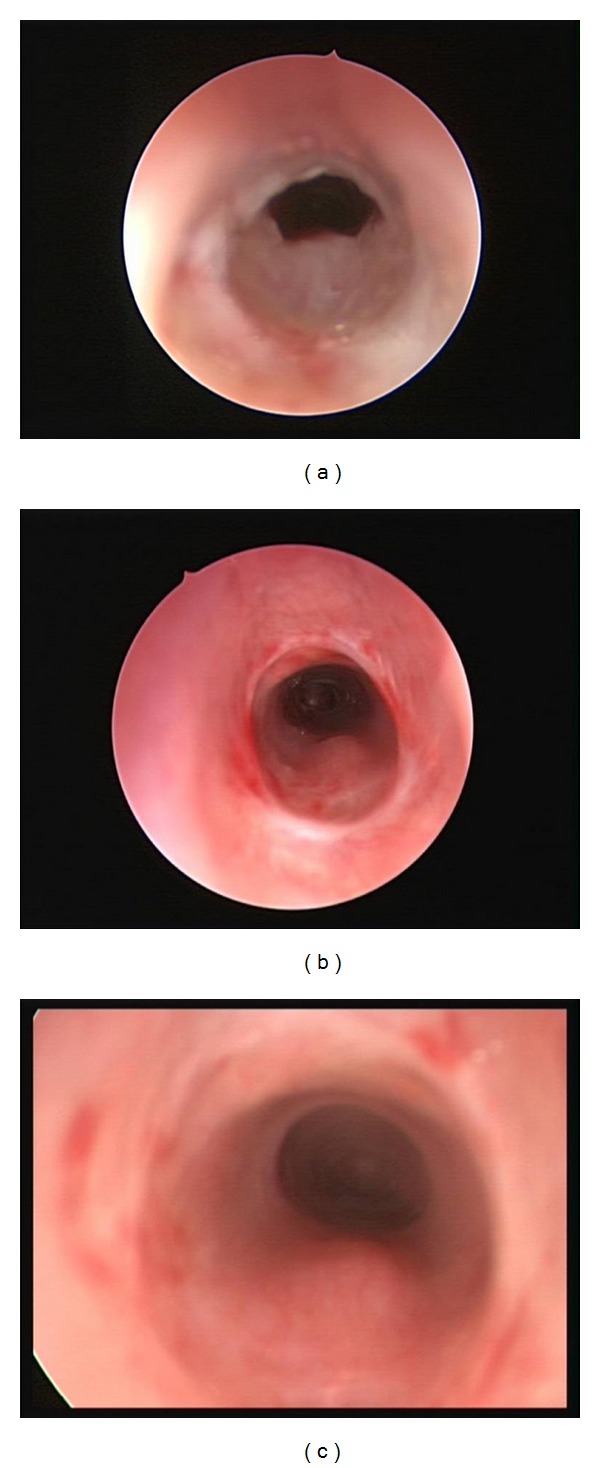
Images of subglottic stenosis at the initial dilation (a), second dilation (b), and 21-month follow-up (c).

Patient 2 was a 14-week-old male who required intubation for 8 days after he presented to emergency department with respiratory failure. He failed extubation twice. Airway examination documented Grade II, soft subglottic stenosis and balloon dilation was performed (Figure 2). Repeat balloon dilations were performed at 13 days and 10 weeks after the initial balloon dilation. The airway was stable 5 months after the first balloon dilation. The child has been followed for 21 months with no stridor and increased work of breathing.

Patient 3 was a 6-month-old male who was born at 28 weeks of gestational age. He required endotracheal intubation for 3 weeks. He was discharged home after an 8-week stay in the intensive care unit. He developed bronchiolitis and croup after discharge. He presented to emergency department with a 1-month history of progressively worsening stridor and increased work of breathing. Assessment of the airway documented Grade II, soft subglottic stenosis, and balloon dilation was performed. Repeat balloon dilations were performed at 3 weeks, 7 weeks, and 10 weeks after the initial balloon dilation. The airway was stable 4 months after the initial balloon dilation. The child has been followed for 20 months with no further stridor or increased work of breathing.

## 3. Discussion

Endoscopic and open surgical approaches have been used with varying results to treat acquired subglottic stenosis in adults and children [3–13]. Balloon laryngoplasty, an endoscopic technique, has been increasingly used as primary or adjuvant option in the treatment of acquired subglottic stenosis since outcomes of balloon dilation in children with laryngotracheal stenosis were reported in 1991 [1]. To date, specific indications for the use of balloon dilation in the treatment of acquired subglottic stenosis have not been established.

Outcomes of primary and secondary balloon dilation for treatment of acquired laryngotracheal stenosis in children have been reported with varying results. Primary balloon dilation allowed avoiding open laryngotracheal reconstruction or tracheotomy in 60% to 100% of children [5, 14, 15]. Secondary balloon dilation, the use of balloon dilation as an adjunct to laryngotracheal reconstruction and endoscopic cricoid split, resulted in decannulation of 50% to 80% of children with acquired laryngotracheal stenosis [14–17]. Presence of concomitant airway lesion, such as tracheomalacia, laryngomalacia, subglottic cyst, vocal cord paralysis, subglottic granulation tissue, or complete tracheal ring, increased the likelihood of treatment failure.

Lack of universally accepted and standardized protocol outlining the number of sessions and optimum delay time in between sessions potentially contributed to the observed differences in success rates. The number of serial dilations ranged from 1 to 5 in previous studies. Serial interval evaluation was made after 1-2 weeks over a period of 3 weeks to 2 months. The need for three or more dilations was predictive of failure and open airway reconstruction was considered [14, 16]. In the present study, we employed serial balloon dilations in 3 patients with acquired subglottic stenosis and evaluated the long-term outcomes of the balloon dilation. All patients had soft subglottic stenosis. The number of serial dilations ranged between 2 and 3 over a period of 2 weeks to 10 weeks. Stable airway was achieved at 3 to 5 months after the balloon dilation. Symptoms did not recur at 10 months to 20 months after balloon dilation in this group of children with Grade II and Grade III, soft, acquired subglottic stenosis. Potential limitations of the present study included small sample size, retrospective nature of the study, and lack of a control group. In conclusion, serial balloon dilation was safe and successful method to manage soft, acquired subglottic stenosis in this group of children. No recurrence was noted in a follow-up more than a year after resolution of symptoms. It is hoped that the present study findings will be used to design larger confirmatory studies assessing the effect of total number of balloon dilations, duration of inflation, and the optimum duration of follow-up after balloon dilation for acquired subglottic stenosis in children.

## References

[1] Hebra A, Powell DD, Smith CD, Othersen HB (1991). Balloon tracheoplasty in children: results of a 15-year experience. *Journal of Pediatric Surgery*.

[2] Myer CM, O’Connor DM, Cotton RT (1994). Proposed grading system for subglottic stenosis based on endotracheal tube sizes. *Annals of Otology, Rhinology and Laryngology*.

[3] Cotton RT, Myer CM (1984). Contemporary surgical management of laryngeal stenosis in children. *The American Journal of Otolaryngology*.

[4] Cohen MD, Weber TR, Rao CC (1984). Balloon dilatation of tracheal and bronchial stenosis. *The American Journal of Roentgenology*.

[5] Durden F, Sobol SE (2007). Balloon laryngoplasty as a primary treatment for subglottic stenosis. *Archives of Otolaryngology*.

[6] Ang AH, Modi VK, Raithatha R, April MM, Ward RF (2010). A pilot study of balloon dilation in an animal model resulting in cricoid cartilage fracture: Implications for the stenotic pediatric airway. *Laryngoscope*.

[7] Walner DL, Loewen MS, Kimura RE (2001). Neonatal subglottic stenosis—incidence and trends. *Laryngoscope*.

[8] Luft JD, Wetmore RF, Tom LWC, Handler SD, Potsic WP (1989). Laryngotracheoplasty in the management of subglottic stenosis. *International Journal of Pediatric Otorhinolaryngology*.

[9] Lando T, April MM, Ward RF (2008). Minimally invasive techniques in laryngotracheal reconstruction. *Otolaryngologic Clinics of North America*.

[10] Lee KH, Rutter MJ (2008). Role of balloon dilation in the management of adult idiopathic subglottic stenosis. *Annals of Otology, Rhinology and Laryngology*.

[11] Collins WO, Kalantar N, Rohrs HB (2012). The effects of balloon dilation laryngoplasty in children with congenital heart disease. *Archives of Otolaryngology*.

[12] Holinger PH, Johston KC (1958). The management of chronic laryngeal stenosis. *Annals of Otology, Rhinology, and Laryngology*.

[13] Campbell BH, Dennison BF, Durkin GE (1986). Early and late dilatation for acquired subglottic stenosis. *Otolaryngology*.

[14] Whigham AS, Howell R, Choi S, Peña M, Zalzal G, Preciado D (2012). Outcomes of balloon dilation in pediatric subglottic stenosis. *Annals of Otology, Rhinology, and Laryngology*.

[15] Bent JP, Shah MB, Nord R, Parikh SR (2010). Balloon dilation for recurrent stenosis after pediatric laryngotracheoplasty. *Annals of Otology, Rhinology and Laryngology*.

[16] Hautefort C, Teissier N, Viala P, Van Den Abbeele T (2012). Balloon dilation laryngoplasty for subglottic stenosis in children: eight years’ experience. *Archives of Otolaryngology*.

[17] Mirabile L, Serio PP, Baggi RR, Couloigner VV (2010). Endoscopic anterior cricoid split and balloon dilation in pediatric subglottic stenosis. *International Journal of Pediatric Otorhinolaryngology*.

